# Multi-compartment diffusion magnetic resonance imaging models link tract-related characteristics with working memory performance in healthy older adults

**DOI:** 10.3389/fnagi.2022.995425

**Published:** 2022-10-05

**Authors:** Christopher E. Bauer, Valentinos Zachariou, Pauline Maillard, Arvind Caprihan, Brian T. Gold

**Affiliations:** ^1^Department of Neuroscience, University of Kentucky, Lexington, KY, United States; ^2^Department of Neurology, University of California at Davis, Davis, CA, United States; ^3^Center for Neuroscience, University of California at Davis, Davis, CA, United States; ^4^The Mind Research Network, Albuquerque, NM, United States; ^5^Sanders-Brown Center on Aging, Lexington, KY, United States

**Keywords:** aging, brain, white matter, diffusion tensor imaging (DTI), free water, neurite orientation dispersion and density imaging (NODDI), working memory, functional networks

## Abstract

Multi-compartment diffusion MRI metrics [such as metrics from free water elimination diffusion tensor imaging (FWE-DTI) and neurite orientation dispersion and density imaging (NODDI)] may reflect more specific underlying white-matter tract characteristics than traditional, single-compartment metrics [i.e., metrics from Diffusion Tensor Imaging (DTI)]. However, it remains unclear if multi-compartment metrics are more closely associated with age and/or cognitive performance than single-compartment metrics. Here we compared the associations of single-compartment [Fractional Anisotropy (FA)] and multi-compartment diffusion MRI metrics [FWE-DTI metrics: Free Water Eliminated Fractional Anisotropy (FWE-FA) and Free Water (FW); NODDI metrics: Intracellular Volume Fraction (ICVF), Orientation Dispersion Index (ODI), and CSF-Fraction] with both age and working memory performance. A functional magnetic resonance imaging (fMRI) guided, white matter tractography approach was employed to compute diffusion metrics within a network of tracts connecting functional regions involved in working memory. Ninety-nine healthy older adults (aged 60–85) performed an in-scanner working memory task while fMRI was performed and also underwent multi-shell diffusion acquisition. The network of white matter tracts connecting functionally-activated regions was identified using probabilistic tractography. Diffusion metrics were extracted from skeletonized white matter tracts connecting fMRI activation peaks. Diffusion metrics derived from both single and multi-compartment models were associated with age (*p*_*s*_ ≤ 0.011 for FA, FWE-FA, ICVF and ODI). However, only multi-compartment metrics, specifically FWE-FA (*p* = 0.045) and ICVF (*p* = 0.020), were associated with working memory performance. Our results suggest that while most current diffusion metrics are sensitive to age, several multi-compartment metrics (i.e., FWE-FA and ICVF) appear more sensitive to cognitive performance in healthy older adults.

## Introduction

Diffusion tensor imaging (DTI), which is based on a single compartment MRI diffusion model, has shown promise as a method for identifying white matter networks associated with specific cognitive functions and how they are influenced by age (Minati et al., 2007; Gunning-Dixon et al., 2009; Madden et al., 2012; Bennett and Madden, 2014). DTI is an *in-vivo* method used to explore white matter microstructural properties by estimating the rate and direction of water diffusion within specific tracts of interest (Basser et al., 1994; Pierpaoli et al., 1996; Le Bihan et al., 2001). One of the most commonly used DTI metrics is fractional anisotropy (FA), which reflects the directional variance of apparent diffusion coefficients and broadly indexes neurite density, neurite orientation distribution and other microstructural properties (Beaulieu, 2009; Wheeler-Kingshott and Cercignani, 2009).

However, DTI metrics such as FA are known to have several limitations including an inability to account for crossing fibers, and partial volume effects, which limit interpretations that can be drawn (Wheeler-Kingshott and Cercignani, 2009). For example, concerning partial volume effects, FA is known to be susceptible to free water contamination, which is particularly salient when studying participants who vary in amount of atrophy and ventricular enlargement. These limitations have led to the development of multi-compartment models that make use of multiple diffusion weightings to separate the diffusion signal into tract-related and free water [or cerebrospinal fluid (CSF)] compartments (Pasternak et al., 2009). Two compartment, free water elimination (FWE) models can yield metrics of free water (FW) and the more white matter tract-related metric of free water eliminated FA (FWE-FA) (Ji et al., 2017; Duering et al., 2018; Maillard et al., 2019, 2022; Bergamino et al., 2021).

More recent three-compartment models estimate additional tract-related metrics. For example, neurite orientation dispersion and density imaging (NODDI) (Zhang et al., 2012) models diffusion data as one of three distinct diffusion patterns (and inferred anatomical compartments): isotropic diffusion (CSF or Free Water compartment), hindered diffusion [extraneurite compartment; space around neurites (i.e., axons and dendrites) including neuronal and glial cell bodies] and restricted diffusion (intraneurite compartment; intra axonal/dendritic space). The resulting metrics derived from NODDI are intracellular volume fraction (representing neurite density), orientation dispersion index (representing angular variation of neurites), and CSF Fraction (representing FW). NODDI metrics have been shown to be highly reproducible (Chang et al., 2015; Lehmann et al., 2021).

Several studies have assessed the potential advantages offered by multi-compartment models, relative to DTI, in predicting age and/or cognitive performance (Cox et al., 2016; Ji et al., 2017; Chad et al., 2018; Duering et al., 2018; Maillard et al., 2019; Beck et al., 2021). Results from these studies have suggested that metrics derived from multi-compartment models may be more sensitive predictors of age (Billiet et al., 2015; Cox et al., 2016; Kodiweera et al., 2016; Chad et al., 2018; Beck et al., 2021) and/or cognitive performance in older adults (Merluzzi et al., 2016; Ji et al., 2017; Duering et al., 2018; Maillard et al., 2019; Fu et al., 2020). Notably, most studies have focused on white matter across the brain (Ji et al., 2017; Chad et al., 2018; Duering et al., 2018; Maillard et al., 2019, 2022; Beck et al., 2021) and/or explored individual white matter tracts that form portions of multiple cognitive networks (Cox et al., 2016; Ji et al., 2017; Chad et al., 2018; Beck et al., 2021).

Less remains known about which diffusion metrics are the most sensitive predictors of age and/or performance within large-scale, task-relevant white matter networks. This issue is relevant in that cognitive functions are known to arise from connected brain networks (Bressler and Menon, 2010; Petersen and Sporns, 2015). Here we address this issue *via* a combined fMRI-guided, DTI tractography approach in which we define a set of white matter tracts inter-connecting functionally activated working memory regions. A working memory fMRI task was used because working memory declines significantly with age and is predictive of later cognitive impairment in older adults (Zacks et al., 2000; Reuter-Lorenz and Sylvester, 2005; Blacker et al., 2007; Glisky, 2007; Belleville et al., 2008). Diffusion metrics from single and multi-compartment models were extracted from the common working memory network of white matter tracts and their associations with age and working memory performance were explored in linear regression models that controlled for shared variance.

## Materials and methods

### Participants

Ninety-nine healthy older adults were recruited for the experiment (61 women, age range 60–85 years). All participants provided informed consent under a protocol approved by the Institutional Review Board of the University of Kentucky. Participants were recruited from an existing longitudinal cohort at the Sanders-Brown Center on Aging (SBCoA) and the Lexington community. Participants from the SBCoA were cognitively intact based on scores from the Uniform Data Set (UDS3) used by US ADRCs [procedure outlined in Besser et al. (2018)] and clinical consensus diagnosis. Participants recruited from the community did not complete the UDS3 battery but were required to score 26 or above on the Montreal Cognitive Assessment (MoCA; Nasreddine et al., 2005) as a study inclusion criteria.

Exclusion criteria were significant head injury (defined as loss of consciousness for more than 5 min), stroke, neurological disorders (e.g., epilepsy, dementia), psychiatric disorders (e.g., schizophrenia, active clinical depression), claustrophobia, pacemakers, the presence of any metal fragments or implants that are incompatible with MRI, diseases affecting the blood (anemia, kidney/heart disease) or significant brain abnormalities detected during imaging. One participant was excluded from analyses due to the presence of an old stroke that was not clinically evident at study enrollment. Detailed characteristics of the final participant cohort are reported in Table 1.

**TABLE 1:** Group demographics and Montreal Cognitive Assessment (MoCA) scores.

	Mean (S.D.)	*N*
Age (Years)	70.0 (5.8)	99
Gender Ratio (F:M)	61:38	99
Education (Years)	16.6 (2.4)	99
MoCA	27.2 (2.2)	93

The table lists the mean (sd) for age, the female/male ratio, and the mean (sd) years of education and MoCA scores.

### Image acquisition

Participants were scanned in a 3 Tesla Siemens Magnetom Prisma MRI scanner (software version E11C), using a 64-channel head coil, at the University of Kentucky’s Magnetic Resonance Imaging and Spectroscopy Center (MRISC). Data from 5 sequences were collected in the following order (1) a 3D multi-echo, T1-weighted magnetization prepared rapid gradient echo (T1) sequence; (2) a T2*-weighted, gradient-echo, echo-planar sequence sensitive to the BOLD response; (3) a double-echo, gradient-echo sequence used to create a field map image for spatial distortion correction of the fMRI data; (4) a spin-echo, echo-planar multi-shell diffusion sequence and (5) a spin-echo, echo-planar diffusion-weighted sequence with reverse phase-encoding direction from the main multi-shell diffusion sequence to correct susceptibility-induced distortions in the main multi-shell diffusion scan. This main multi-shell diffusion sequence is also collected as part of our Alzheimer’s Disease Neuroimaging Initiative (ADNI3; Gunter et al., 2017) scanning protocol. Data from several other sequences were collected during the scanning session related to different scientific questions and are not discussed further here.

The T1 scan had four echoes [first echo time (TE1) = 1.69 ms, echo spacing (ΔTE = 1.86 ms)], and covered the entire brain [256 × 256 × 176 mm^3^ acquisition matrix (176 slices), 1 mmisotropic voxels, repetition time (TR) = 2530 ms, flip angle = 7°, scan duration = 5.88 min]. Two fMRI runs were acquired and covered the entire brain [192 × 192 × 120 mm^3^ acquisition matrix (40 slices), 3 mm isotropic voxels, TR = 2500 ms, TE = 30 ms, flip angle = 90°, scan duration = 4.12 min per run]. The gradient echo field map scan was acquired immediately after the two fMRI runs [TR = 450 ms, TE_1_ = 5.19 ms, TE_2_ = 7.65 ms, flip angle = 60°, scan duration = 1.23 min] and had the same field of view, number of axial slices, and resolution as the fMRI scans. The main multi-shell diffusion scan was acquired with 126 separate diffusion directions [232 × 232 × 162 mm^3^ acquisition matrix (81 slices), 2 mm isotropic voxels, TR = 3400 ms, TE = 71 ms, simultaneous multislice acceleration factor = 3, phase partial Fourier = 6/8, scan duration = 7.45 min, and posterior-to-anterior phase encoding direction] distributed among 4 b-values [0 s/mm^2^ (12 directions), 500 s/mm^2^ (6 directions), 1000 s/mm^2^ (48 directions), and 2000 s/mm^2^ (60 directions)]. The brief (28 s) reverse-phase encoding (anterior-to-posterior) scan was acquired immediately following the main multi-shell diffusion scan [232 × 232 × 162 mm^3^ acquisition matrix (81 slices), 2 mm isotropic voxels, TR = 3400 ms, TE = 71 ms, simultaneous multislice acceleration factor = 3, phase partial Fourier = 6/8, and 2 b-values (0 and 2000 s/mm^2^)]. Only the non-diffusion weighted (b0) images were used to correct for susceptibility-induced distortions in the main multi-shell diffusion scan, as recommended by FSL’s topup (Andersson et al., 2003). These MRI sequences are further summarized in Supplementary Table 1.

### Functional magnetic resonance imaging task paradigm

The visual working memory paradigm used in the current study is the same as the one described in detail in Zachariou et al. (2021). Briefly, participants performed an N-back task with 3 conditions (compare, 1-back, 2-back). A blocked design was used with each of the 3 conditions interleaved within two separate fMRI runs. Task stimuli consisted of eight consonant letters and responses (“same” or “different”) were made using MRI compatible button-boxes (one in each hand). For the working memory conditions of the task (1-back, 2-back) participants decided if the consonant letter presented in the current trial matched the one presented in the previous trial (1-back condition) or two trials back (2-back condition). For the control condition (compare) participants decided if two consonant letters presented simultaneously on either side of the screen were the same or different.

#### Behavioral data analysis

Behavioral data collected during the scans were used to calculate D-prime (Stanislaw and Todorov, 1999) for each of the task conditions. D-prime is a measure of discrimination performance corrected for response bias, which is the participants’ tendency to respond “same” or “different” when they do not perceive a difference or are not certain. Therefore, in forced-choice discrimination tasks, D-prime is a more optimal measure of discrimination performance than accuracy, which does not account for response bias (Swets et al., 1961). D-prime was log transformed in all analyses due to a non-normal distribution and the assumption that diffusion-based measures will logarithmically predict D-prime, as done in Zachariou et al. (2021). N-back task performance was ultimately expressed as the log of averaged D-prime (averaged across the 1-back and 2-back conditions).

#### Defining the functional magnetic resonance imaging working memory network

The fMRI data were corrected for field inhomogeneity using the field map in FMRIB software library (FSL; Smith et al., 2004), were motion-corrected, and were despiked when required using AFNI (3dDspike; Cox, 1996). The fMRI data were then aligned to the structural T1 scan (after the 4 echoes from the T1 scan were averaged into a single root mean squared image) and warped into MNI space (MNI ICBM152 1 mm 6th generation atlas; Grabner et al., 2006) *via* non-linear transformation (3dQwarp; Cox, 1996). Finally, the fMRI data were smoothed with a 6.0 mm full width at half maximum Gaussian kernel and mean-based intensity normalized using AFNI (Cox, 1996).

**FIGURE 1:**
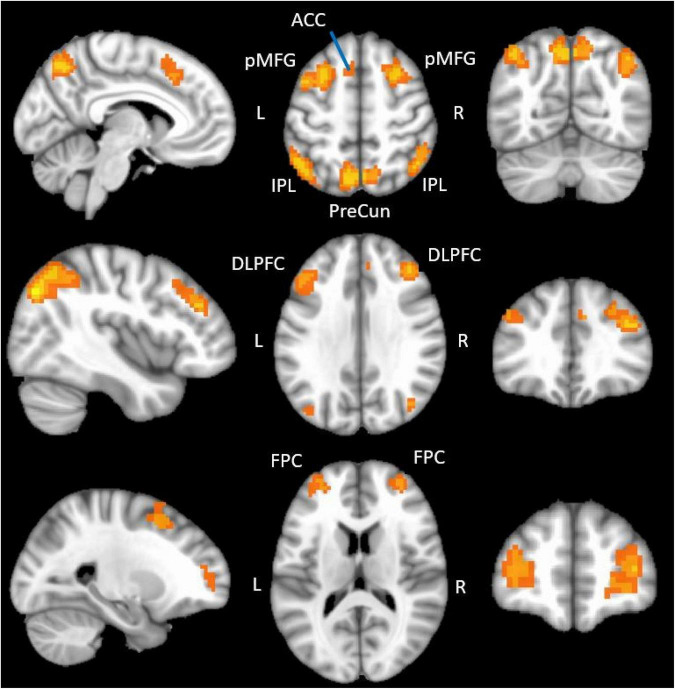
fMRI regions showing functional activation during the N-back tasks. The figure displays the positive functional activation using a group level functional contrast [(2-back/2 + 1-back/2) > Compare] overlaid onto a 1 mm MNI ICBM152 template (MNI ICBM152 1 mm 6th generation atlas; Grabner et al., 2006). fMRI data were thresholded (q = 1 × 10^– 10^) and clusterized (minimum cluster size = 20) in this figure to optimize display. ACC, anterior cingulate; pMFG, posterior middle frontal gyrus; IPL, inferior parietal lobule; PreCun, precuneus; DLPFC, dorsolateral prefrontal cortex; FPC, frontopolar cortex.

Group-level, whole brain analyses were then conducted using AFNI and a linear mixed effects model (3dLME; Chen et al., 2013) with participant age added as a covariate. Specifically, this analysis used a whole-brain functional contrast [(1-back/2 + 2-back/2) > Compare] to identify brain regions significantly more active during the 1-back and 2-back conditions of the working memory task than during the compare control condition. The resulting activation map was adjusted for multiple comparisons using the false discovery rate (FDR) approach (Figure 1). A conservative threshold of qFDR = 2 × 10^–13^ was used to limit activations to the most significantly active voxels in order to delineate distinct peaks of activity for use as seed regions in a subsequent probabilistic tractography analysis. Twelve brain regions were identified using this threshold and are reported in Table 2. Lastly, an 8 mm diameter sphere was centered on the most statistically significant voxel of each of these twelve brain regions. These 8 mm diameter spheres acted as seed regions of interest (ROIs) in a subsequent probabilistic tractography analysis (Section “Probabilistic tractography”).

**TABLE 2:** fMRI regions showing functional activation during the N-back tasks.

Anatomical region	Hemisphere	Peak coordinates
		(X, Y, Z)
Frontopolar cortex	L	−33, 52, 6
Frontopolar cortex	R	32, 55, 8
Dorsolateral prefrontal cortex	L	−44, 29, 33
Dorsolateral prefrontal cortex	R	41, 37, 30
Posterior middle frontal gyrus	L	−26, 12, 52
Posterior middle frontal gyrus	R	28, 13, 52
Anterior inferior parietal lobule	L	−43, −59, 50
Anterior inferior parietal lobule	R	44, −60, 49
Posterior inferior parietal lobule	L	−34, −77, 42
Posterior inferior parietal lobule	R	42, −72, 37
Anterior cingulate cortex	L	−6, 24, 43
Precuneus	L	−7, −68, 52

The table lists the regions that were most significantly active during the working memory (1-back and 2-back) conditions compared to the control condition (functional contrast of [2-back/2 + 1-back/2] > Compare). The peak coordinates are reported in MNI space (LPI/SPM) and refer to the voxel with the greatest positive activation.

### Diffusion magnetic resonance imaging preprocessing

Diffusion MRI data were preprocessed as follows: each participant’s main diffusion MRI data were corrected for susceptibility induced field distortions using their reversed phase-encoded scan in FSL’s topup (Andersson et al., 2003), skull-stripped using BET (Smith, 2002), and non-linearly corrected for eddy currents and participant motion with eddy (Andersson and Sotiropoulos, 2016) using the Compute Unified Device Architecture (CUDA; version 9.1) command variant (eddy_cuda9.1) to increase data analysis processing speed. Diffusion MRI data were examined visually for quality. The average head motion across volumes for each participant was assessed using the eddy QC tools (average voxel displacement across all voxels within a brain mask relative to the first volume; Bastiani et al., 2019), with a 2 mm threshold used for exclusion. No participants exceeded this threshold. Each participant’s motion-corrected data were subsequently used as input for further diffusion processing and probabilistic tractography.

### Diffusion tensor imaging processing

Fractional anisotropy (FA) maps were calculated using FSL’s DTIFIT. This function computes the diffusion tensor model and eigenvalues (λ_1_, λ_2_, λ_3_) within each voxel using each participant’s preprocessed diffusion MRI data as described in Section “Diffusion magnetic resonance imaging preprocessing.”

#### Free water elimination processing

Both free water [FW; representing cerebrospinal fluid (CSF)] and free water eliminated (FWE, or CSF-eliminated) FA maps were calculated for each participant using a two-compartment model of the multi-shell Free Water Diffusion Tensor Imaging algorithm (Henriques et al., 2017) from the open-source software package Diffusion Imaging in Python (DIPY; Garyfallidis et al., 2014). All voxels with values above the 80th percentile of values in the CSF/FW compartment map were set to zero in the corresponding FWE-FA map, in order to suppress any remaining CSF signal (e.g., within the ventricles) that could contribute to partial volume effects (Zhang et al., 2012).

#### Neurite orientation dispersion and density processing

Neurite orientation dispersion and density imaging employs a three-compartment model which distinguishes between isotropic diffusion (CSF or FW compartment), hindered diffusion (extraneurite compartment) and restricted diffusion (intraneurite compartment) (Zhang et al., 2012). Three diffusion metrics are subsequently calculated per-voxel using these compartments. Intracellular volume fraction (ICVF) is the intraneurite compartment divided by the sum of the intraneurite and extraneurite compartments, and represents neurite density (Zhang et al., 2012). Orientation dispersion index (ODI) is the angular variation in neurite orientation, and CSF Fraction (FW) is the CSF/FW compartment divided by the sum of all three compartments. All three metric maps were calculated for each participant using the default settings from the NODDI MATLAB toolbox [toolbox version 1.04 (Zhang et al., 2012); MATLAB version R2019 Update 7]. As done in the free water elimination model, any voxels that were above an 80% threshold in the CSF Fraction/FW map were set to zero in the corresponding ICVF or ODI maps to reduce partial volume effects (Zhang et al., 2012).

### Probabilistic tractography

Estimates of anatomical, white-matter connectivity between the 12 seed ROIs defined in Section “Defining the fMRI working memory network” were calculated using probabilistic tractography in FSL, as described in our previous work (Brown et al., 2015, 2017). First, the CUDA/GPU version of BEDPOSTX (BEDPOSTX_GPU) was used to construct per participant maps of the distribution of diffusion parameters at each voxel, from the eddy corrected diffusion data (described in Section “Diffusion magnetic resonance imaging preprocessing”) as input (Behrens et al., 2007). The distribution of diffusion parameters was modeled using zeppelins (model 3).

Next, each participant’s high resolution T1 image was aligned to a b0 image from their motion-corrected diffusion MRI data (Section “Diffusion magnetic resonance imaging preprocessing”) using the AFNI function align_epi_anat.py and a local Pearson correlation cost function. The aligned T1 image was then non-linearly warped to MNI152 space (MNI ICBM152 1 mm 6th generation atlas; Grabner et al., 2006) using the AFNI function auto_warp.py. Finally, the inverse of the transformation matrix obtained in the previous step was used to warp the 12 seed gray matter fMRI ROIs (defined in Section “Defining the functional magnetic resonance imaging working memory network”), and a brainstem mask created from the Harvard-Oxford Subcortical Atlas (Desikan et al., 2006), from MNI152 space to each participant’s native diffusion MRI space using 3dNwarpApply and a nearest neighbor cost function. The brainstem mask was used to constrain the probabilistic tractography step described below.

The 12 native-space-warped seed ROIs obtained previously were used as inputs to the CUDA/GPU version of FSL’s PROBTRACKX2 (PROBTRACKX2_GPU) in order to calculate estimates of anatomical connectivity between the seed ROIs (Behrens et al., 2003, 2007). PROBTRACKX2_GPU was executed using modified Euler streamlining in network mode. Five-thousand streamlines were generated from each voxel within each of the seed ROIs, with a maximum of 2,000 steps per streamline, a step length of 0.5 mm, a minimum streamline length of 20 mm, a curvature threshold of 0.2 (curvature angle could not exceed approximately 80°), and the default fiber volume threshold of 0.01. To prevent tracking of streamlines across the brainstem, the native-space-warped brainstem mask (obtained in the previous step), was used as an exclusion mask as described in our previous work (Brown et al., 2015). Tracking between seed ROIs for any streamline stopped if any of the following failure criteria were met: streamlines extending from one seed ROI did not reach any of the other seeds, the minimum streamline length requirements were not met, the streamline curvature threshold was exceeded (greater than 80°), the streamline exited the brain, or looped back on itself, or the streamline entered the exclusion mask (brainstem).

The output of PROBTRACKX2 is a streamline density map, containing successful streamlines per voxel for each participant. A proportion image was then created for each participant by dividing each streamline density map by the sum of all voxels across the seed ROIs within that participant, to correct for any potential differences in the total number of streamlines generated between participants. Therefore, each participant’s proportion image provides a quantitative measure of the proportion of successful streamlines that passed through each voxel. Next, each participant’s proportion image was divided by the waytotal, which is the sum of all the successful streamlines for that participant, to account for differences in the “ease of tractability” across all participants (Brown et al., 2015).

### Tract-based spatial statistics

Each participant’s FA map was transformed into standard space and skeletonized using FSL’s tract-based spatial statistics (TBSS) pipeline (Smith et al., 2006), as described in our previous work (Bauer et al., 2021). After an initial preprocessing step (tbss_1_preproc), non-linear voxel-wise registration was used to transform each participant’s FA image into 1mm FMRIB58_FA space. These transformed images were then averaged to create a mean FA image (tbss_2_reg and tbss_3_postreg), from which a common white matter tract skeleton was created. All participants FA data was then projected onto this skeleton [i.e., “skeletonized”; thresholded at FA > 0.2 (tbss_4_prestats)] to correct partial volume effects that may occur after warping. All other diffusion metric maps (Section “Diffusion tensor imaging processing”; FW, FWE-FA, ICVF, ODI, CSF Fraction) from each participant were likewise processed with the same templates, transformations, and skeletonization using FSL’s tbss_non_FA pipeline. Each participant’s waytotal-normalized proportion images (Section “Probabilistic tractography”) were also processed with the same templates and transformations using tbss_non_FA, but were not skeletonized. All participants non-skeletonized waytotal-normalized proportion images in FMRIB58_FA space were used to produce a group working memory network white matter (WMN-WM) mask (Section “Extracting diffusion metrics from the group WMN-WM mask”).

#### Extracting diffusion metrics from the group WMN-WM mask

**FIGURE 2:**
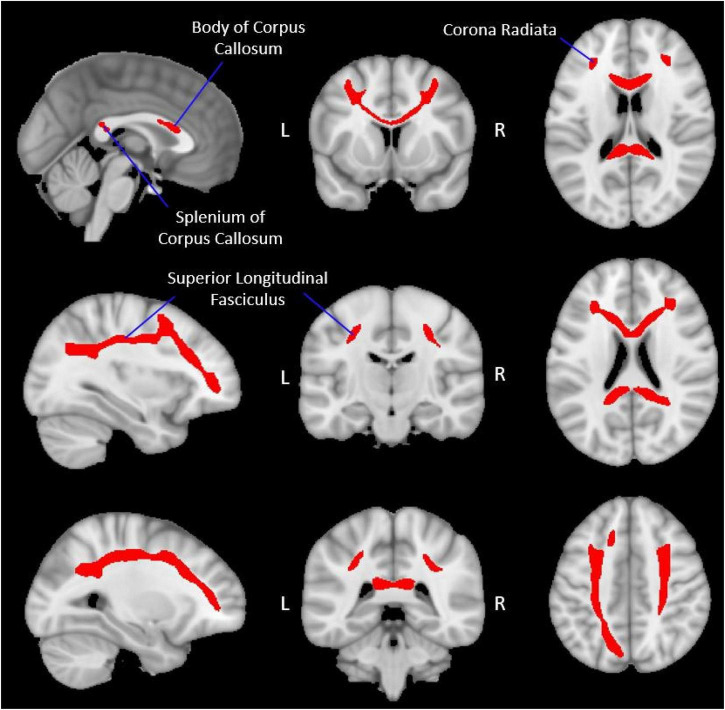
Group-level working memory network white matter mask. The figure displays the group-level white matter tracts (red), identified *via* probabilistic tractography, which make up the WMN-WM mask (Section “Extracting diffusion metrics from the group WMN-WM mask”) overlaid onto a 1 mm MNI ICBM152 template (MNI ICBM152 1 mm 6th generation atlas; Grabner et al., 2006). Peak regions of activation during the fMRI working memory task were used as seed regions (Table 2 and Figure 1) to create this WMN-WM mask.

A group working memory network white matter (WMN-WM) mask was created by averaging each participant’s waytotal-normalized proportion image into a group mean image (Figure 2; Supplementary Figure 1) and applying a percentile threshold to include only the voxels with values in the top 3% using AFNI’s 3dttest++ and a clusterization procedure (3dclust; minimum cluster size = 100) to remove any voxels that survived the threshold but were not connected to the primary WMN-WM mask. Several other threshold values (top 2%, top 5%, top 10%) were also explored but yielded poorer results (Supplementary Tables 2, 3 and Supplementary Figure 2), and produced a network which was either too restrictive (top 2%) or less specific (capturing white matter less relevant to working memory; top 5% and top 10%).

Each participant’s skeletonized diffusion metric maps (FA, FW, FWE-FA, ICVF, ODI, CSF Fraction; Section “Tract-based spatial statistics”) were then restricted to voxels overlapping with the binarized group WMN-WM mask and the mean of non-zero voxels was extracted from each participant’s maps using FSL (fslmeants).

### Statistical analyses

Statistical analyses were conducted using SPSS 27 (IBM, Chicago, IL, USA). In our initial analyses we compared both mean D-prime and mean log D-prime between the compare and N-back (averaged across the 1-back and 2-back conditions) conditions using a paired-sample *t*-test. For our fMRI-diffusion MRI analyses, we first report the network of brain regions showing functional BOLD activation during the working memory task (Figure 1 and Table 2) and the white matter tracts connecting these functionally activated brain regions (Figure 2). Subsequent analyses explored how strongly each diffusion metric within the identified white matter tracts was associated with age and with log D-prime from the N-back tasks (Section “Behavioral data analysis”). As mentioned in Section “Extracting diffusion metrics from the group WMN-WM mask,” we thresholded the group mean waytotal-normalized proportion image to include only those voxels with values in the top 3%, but other thresholds were tested both qualitatively (Supplementary Figure 2) and for quantitative assessment with age (Supplementary Table 2) and cognition (Supplementary Table 3).

A single model with all diffusion metrics predicting age could not be employed as values from several diffusion metrics were correlated and overlapped in the intended diffusion measurement. For this reason, three conceptually-grouped models were used to predict age. The first model was based on the single-compartment DTI data, where only standard FA in the working memory network white matter (WMN-WM) regions was used as a predictor of age. The second model was grouped based on the two-compartment free water elimination model, with both the FWE-FA and FW in WMN-WM regions included as predictors. The final model was grouped based on the three-compartment NODDI model, with ICVF, ODI, and CSF Fraction in WMN-WM regions included as the predictors. In all three models, sex was used as a covariate.

The same strategy of grouping predictors was used in three models predicting log D-prime during the N-back tasks (Model 1; FA, Model 2; FW and FWE-FA, Model 3; ICVF, ODI, and CSF Fraction). In these three models, both age and sex were used as covariates.

Results were considered statistically significant at *p* < 0.05. Statistical outliers were defined as values greater than 3 standard deviations from the group mean and were excluded from relevant analyses. Error residuals in all linear regression models were examined for the assumption of normality using Q-Q plots (Supplementary Figure 3). The variance inflation factor (VIF) between predictors in linear regression models was not permitted to exceed a value of 5 (Stine, 1995) to limit the effects of collinearity.

## Results

### Participant and data characteristics

Participant summary demographics are presented in Table 1 (Section “Participants”). Error residuals in all linear regression models followed a normal distribution (Supplementary Figure 3). In all linear regression models, the VIF for all predictors was less than 2 and tolerance was greater than 0.5.

### N-back task performance

D-prime data from one participant were unavailable and data from another participant had one D-prime outlier value, which was removed from relevant analyses (Sections “N-back task performance” and “Association between diffusion metrics and working memory performance”; *N* = 97 for Section “N-back task performance”). Mean D-prime across participants was 6.43 in the compare condition and 3.45 in the averaged 1-back and 2-back conditions, which was a significant difference (*t* = 14.138, *p* < 0.001). Participants also had a significantly higher log D-prime score in the compare condition than in the averaged 1-back and 2-back conditions (*t* = 10.914, *p* < 0.001), as expected.

### Functional magnetic resonance imaging working memory network

We identified twelve regions significantly more active in the N-back conditions than the compare control condition. These included bilateral dorsolateral prefrontal cortices, posterior middle frontal gyri, frontopolar cortices, anterior inferior parietal lobules, posterior inferior parietal lobules, left anterior cingulate cortex and left precuneus (Figure 1 and Table 2). The peak coordinates from all positively active regions (Table 2) were used as inputs in the Neurosynth database (Yarkoni et al., 2012), which revealed that our peaks directly overlapped with, or were immediately adjacent to, core working memory network regions identified using a meta-analysis of 1,334 working memory-related studies.

### Probabilistic tractography

The white matter tracts connecting the fMRI-defined working memory network included the body and splenium of the corpus callosum, bilateral portions of the superior longitudinal fasciculus, and bilateral portions of the corona radiata (Figure 2). The frontal and parietal fMRI seeds are interconnected with their contralateral homologues primarily through the corpus callosum while the ipsilateral frontal and parietal fMRI seeds are primarily interconnected through the superior longitudinal fasciculus.

### Association between diffusion metrics and age

Data from two participants were excluded from the Sections “Association between diffusion metrics and age” and “Association between diffusion metrics and working memory performance” due to visible artifacts in diffusion data and the presence of outlier values in calculated diffusion metrics (*N* = 97 for Section “Association between diffusion metrics and age” and *N* = 95 for Section “Association between diffusion metrics and working memory performance”). All diffusion metrics in the Sections “Association between diffusion metrics and age” and “Association between diffusion metrics and working memory performance” were computed from skeletonized tracts within the WMN-WM mask (Section “Extracting diffusion metrics from the group WMN-WM mask”; Figure 2). FA was negatively correlated with age (β = −0.425; *p* < 0.001) in the first model (Table 3). In the second model, FWE-FA was negatively associated with age (β = −0.407; *p* = 0.001), while FW was not associated with age (β = 0.069; *p* = 0.570). In the final model, ICVF was negatively associated with age (β = −0.377; *p* < 0.001), ODI was positively associated with age (β = 0.247; *p* = 0.011), and CSF Fraction was not associated with age (β = 0.140; *p* < 0.137).

**TABLE 3:** Summary of linear regression models with diffusion metrics extracted from the WMN-WM mask predicting age.

Diffusion metric	Standardized beta	*T*-value	*P*-value
**Model 1**			
FA	–0.425	–4.518	<0.001*
**Model 2**			
FW	0.069	0.570	0.570
FWE-FA	–0.407	–3.348	0.001*
**Model 3**			
ICVF	–0.377	–4.029	<0.001*
ODI	0.247	2.611	0.011*
CSF fraction	0.140	1.499	0.137

FA, fractional anisotropy; FW, free water; FWE-FA, free water eliminated fractional anisotropy; ICVF, intracellular volume fraction; ODI, orientation dispersion index; CSF Fraction, cerebrospinal fluid fraction.

*p < 0.05.

### Association between diffusion metrics and working memory performance

**FIGURE 3:**
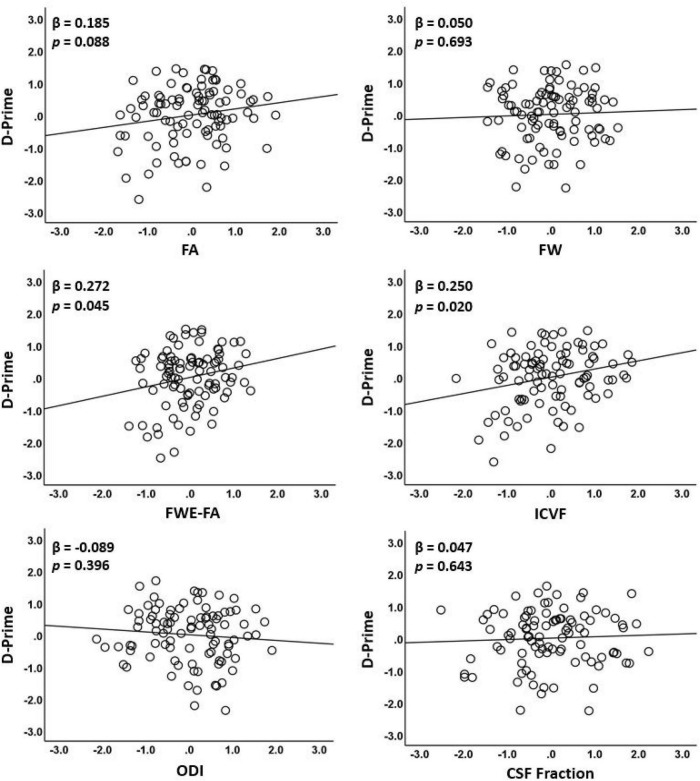
Associations between diffusion metrics extracted from the WMN-WM mask and working memory performance. The figure displays scatterplots of each diffusion metric against log D-prime during the N-back tasks. Only ICVF and FWE-FA were significantly associated with log D-prime (middle row).

Fractional anisotropy was only marginally correlated with D-prime (β = 0.185; *p* = 0.088) in the first model (Table 4 and Figure 3). In the second model, FWE-FA was positively associated with D-prime (β = 0.272; *p* = 0.045), but FW was not associated with D-prime (β = 0.050; *p* = 0.693). In the final model, ICVF was positively associated with D-prime (β = 0.250; *p* = 0.020), while ODI (β = −0.089; *p* = 0.396) and CSF Fraction (β = 0.047; *p* = 0.643) were not associated with D-prime.

**TABLE 4:** Summary of linear regression models with diffusion metrics extracted from the WMN-WM mask predicting working memory performance.

Diffusion metric	Standardized beta	*T*-value	*P*-value
**Model 1**			
FA	0.185	1.724	0.088
**Model 2**			
FW	0.050	0.396	0.693
FWE-FA	0.272	2.034	0.045*
**Model 3**			
ICVF	0.250	2.360	0.020*
ODI	–0.089	–0.852	0.396
CSF fraction	0.047	0.465	0.643

FA, fractional anisotropy; FW, free water; FWE-FA, free water eliminated fractional anisotropy; ICVF, intracellular volume fraction; ODI, orientation dispersion index; CSF Fraction, cerebrospinal fluid fraction.

*p < 0.05.

## Discussion

We compared the strength of association between MRI diffusion metrics derived from single-compartment and multi-compartment models with age and working memory performance in older adults. Comparisons between diffusion metrics were performed within a targeted working memory network of white matter tracts interconnecting functionally activated working memory regions. Our results indicate that FA and most multi-compartment diffusion metrics were strong predictors of age. However, only multi-compartment metrics [FWE-FA and ICVF (neurite density)] were associated with working memory performance. Our results suggest that while FA is a robust predictor of age-related alterations, multi-compartment diffusion metrics appear to be more sensitive predictors of cognitive performance in older adults.

We first identified a set of brain regions showing strong functional response during an N-Back working memory task. The peak positive activations either directly overlapped with, or were immediately adjacent to, peaks identified in a meta-analysis of 1,334 working memory-related studies in the Neurosynth database (Yarkoni et al., 2012). Probabilistic tractography was then used to identify a white matter network composed of tracts connecting the fMRI-defined visual working memory network. The white matter tracts connecting our visual working memory network included portions of the body and splenium of the corpus callosum, bilateral portions of the superior longitudinal fasciculus (SLF) and corona radiata. These tracts have been shown to support working memory and other executive function tasks (Kennedy and Raz, 2009; Madden et al., 2009; Zahr et al., 2009; Gold et al., 2010). For example, the SLF forms a key portion of the lateral frontoparietal network, connecting the DLPFC with the IPL, and contributing to working memory function (Menon, 2011) while the corpus callosum connects the DLPFC and IPL with their contralateral homologues.

### Diffusion metrics and age

Our results demonstrated that FA, FWE-FA, ICVF, and ODI values within a network of tracts supporting working memory were all significant predictors of age. Previous results have shown that conventional DTI metrics such as FA (Lebel et al., 2012; Cox et al., 2016; Chad et al., 2018; Beck et al., 2021), two-compartment metrics of FWE-FA and FW (Chad et al., 2018), and three-compartment NODDI metrics [ICVF, ODI, and CSF Fraction (Billiet et al., 2015; Cox et al., 2016; Kodiweera et al., 2016; Beck et al., 2021)] are all significant predictors of age throughout the majority of white matter tracts studied. Our findings add to this literature by showing that the majority of single and multi-compartment models tested were negatively associated with age within a task-relevant working memory network, even after controlling for shared variance between metrics in our linear regression models.

### Diffusion metrics and working memory performance

In contrast to our findings concerning age, our results indicated that diffusion metrics extracted from a task-relevant white matter network performed differently in predicting working memory performance. FA was significantly associated with working memory performance only after FWE was applied [FA (β = 0.185; *p* = 0.088); FWE-FA (β = 0.272; *p* = 0.045)]. In previous studies exploring only uncorrected FA, age-related reductions in FA have been found to contribute to poorer working memory or related executive function performance in some studies including our own (Charlton et al., 2006, 2010; Gold et al., 2010; Bourbon-Teles et al., 2021) while other studies have reported null results with uncorrected FA (Edde et al., 2020; Gullett et al., 2020). In addition, uncorrected FA is often associated with poorer performance on only a portion of the executive function tasks explored (Grieve et al., 2007; Kennedy and Raz, 2009).

These mixed findings likely in part reflect that traditional DTI measures such as FA are contaminated by FW partial volume effects, particularly in studies with older adults, in whom atrophy and ventricular enlargement are the norm (Pasternak et al., 2009; Timmers et al., 2016). The limited sensitivity of traditional DTI measures (FA) may also reflect that FA is strongly affected by crossing fibers and inherently conflates morphological features such as neurite orientation dispersion and neurite density (Wheeler-Kingshott and Cercignani, 2009; Szczepankiewicz et al., 2015).

We further explored the association between working memory performance and NODDI-derived metrics. In a model including all three NODDI metrics (ICVF; modeling neurite density, ODI; modeling angular variability in neurite orientation, and CSF Fraction; modeling free water), only ICVF was associated with working memory performance (*p* = 0.020). CSF fraction was again not associated with cognitive performance (*p* = 0.643), in agreement with the free water elimination model findings (*p* = 0.693). These cross-sectional results suggest that neurite density may be a better predictor of task performance in healthy older adults than either neurite orientation dispersion (ODI) or free water (FW/CSF Fraction). Additional work which explores multiple cognitive tasks will be needed to explore this possibility.

### Free water was not associated with age or working memory performance

Our results demonstrated that FW was not a significant predictor of age or cognition (in Model 2 or 3), contrasting with some previous reports (Cox et al., 2016; Ji et al., 2017; Maillard et al., 2019; Gullett et al., 2020; Beck et al., 2021). This apparent discrepancy may be explained by several factors. First, in contrast with other studies, we specifically investigated a distributed network of tracts supporting working memory. While FW in global white matter is associated with decreases in cognitive performance (Ji et al., 2017; Duering et al., 2018; Maillard et al., 2019), our results suggest that FW in a defined working memory network is not strongly associated with working memory performance specifically. Second, our study is unique in that the shared variance between predictors in each multi-compartment model was accounted for in subsequent linear regression models (Models 2 and 3). Indeed, FW (from the FWE model) was significantly associated with age (*p* = 0.001) when shared variance was not controlled (Supplementary Table 4), suggesting that FWE-FA accounts for more unique variance than FW as a predictor of age in our participant sample.

However, in our study FW was not associated with working memory performance even when it was entered as a sole model predictor (Supplementary Table 5). Free water quantifies highly isotropic diffusion, which is thought to primarily reflect extracellular water content (Pasternak et al., 2009, 2012; Maillard et al., 2019, 2022). Recent MRI studies further suggest that FW may be a biomarker of global cerebral injury, as elevated FW is associated with a variety of conditions including schizophrenia (Pasternak et al., 2015; Lyall et al., 2018), Alzheimer’s disease (Ji et al., 2017; Bergamino et al., 2021), Parkinson’s disease (Ofori et al., 2015; Planetta et al., 2016), and cerebral small vessel disease/vascular pathology (Ji et al., 2017; Maillard et al., 2017, 2019; Duering et al., 2018). While the exact underlying processes are currently unknown (Maillard et al., 2019), FW is thought to represent relatively advanced damage associated with more global neuroinflammation, neurodegeneration, and/or vascular dysfunction (Ji et al., 2017; Maillard et al., 2017, 2019). Therefore, our finding that FW was not associated with working memory performance may relate to our participant group, which consisted of cognitively normal older adults likely to have less neurodegeneration and vascular pathology than the patient groups described above.

Strengths of our study include the mapping of the white matter connections between fMRI-defined working memory network regions using probabilistic tractography, the use of advanced multi-compartment modeling enabled by multi-shell diffusion imaging, and the consideration of previously validated diffusion metrics (Chang et al., 2015; Albi et al., 2017; Lehmann et al., 2021; Maillard et al., 2022) as predictors of age and cognition. Of particular note, in contrast to most published studies, multiple diffusion metrics were included in the same linear regression models when appropriate (Model 2: FWE-FA and FW; Model 3: ICVF, ODI, and CSF Fraction) to account for shared variance between predictors. Finally, we recruited a moderately large sample size of older adults with a wide age range (60–85 years), and the use of standardized processing and analysis pipelines (AFNI, FSL, TBSS) more easily permits replication.

### Limitations

There are certain limitations to the current study that highlight the need for follow-up investigation. First, our cross-sectional study cannot determine how each diffusion metric might predict declines in working memory performance. Additional longitudinal work is needed to address this issue. Second, as mentioned earlier, our study focused on cognitively normal older adults and it is possible that our findings may not generalize to individuals with more neurodegenerative or cerebrovascular pathology. In particular, some multi-compartment models such as NODDI can have limitations which are exacerbated in the presence of pathology (Lampinen et al., 2017; Kamiya et al., 2020). Future studies should take this into consideration when exploring additional subsets of older adults with elevated dementia or vascular disease risk. Third, our study focused solely on one cognitive domain, working memory. Future studies should compare the sensitivity of diffusion metrics to other cognitive domains, preferably controlling for their shared variance when appropriate.

Finally, free water and NODDI metrics are only a subset of advanced diffusion metrics developed to better model white matter microstructure. Since the primary goal of the present study was to specifically investigate whether multi-compartment diffusion metrics better predict age and cognition than traditional DTI metrics, rather than to compare and contrast all diffusion metrics, many advanced diffusion metrics were inevitably excluded. Further studies are needed to determine the associations between other advanced diffusion metrics, such as metrics from WM tract integrity (WMTI; Fieremans et al., 2011), spherical mean technique (SMT; Kaden et al., 2016), or diffusional kurtosis imaging (DKI; Jensen et al., 2005) with age and cognitive performance.

## Conclusion

Our results suggest that most MRI diffusion metrics derived from current single and multi-compartment models contribute unique variance in the prediction of age. In contrast, our results also suggest that working memory performance in older adults is more specifically associated with tract-related white matter characteristics modeled by multi-compartment diffusion models (i.e., FWE-FA and ICVF). Future longitudinal studies replicating these findings in other older adult samples are needed to confirm this possibility.

## Data availability statement

The raw data supporting the conclusions of this article will be made available by the authors, without undue reservation.

## Ethics statement

The studies involving human participants were reviewed and approved by Institutional Review Board of the University of Kentucky. The patients/participants provided their written informed consent to participate in this study.

## Author contributions

CB: conceptualization, methodology, formal analysis, investigation, data curation, writing—original draft, review and editing, and visualization. VZ: conceptualization, methodology, software, formal analysis, data curation, and writing—review and editing. PM: software, resources, and writing—review and editing. AC: software and writing—review and editing. BG: conceptualization, methodology, formal analysis, resources, data curation, writing—review and editing, visualization, supervision, project administration, and funding acquisition. All authors contributed to the article and approved the submitted version.

## Abbreviations

MRI: magnetic resonance imaging
DTI: diffusion tensor imaging
FA: fractional anisotropy
FWE: free water elimination
NODDI: neurite orientation dispersion and density imaging
ICVF: intracellular volume fraction
ODI: orientation dispersion index
WMN-WM: working memory network white matter.
