# Supplementary material for: Multi-compartment diffusion magnetic resonance imaging models link tract-related characteristics with working memory performance in healthy older adults
Source: Front Aging Neurosci. 2022 Oct 5;14:995425. doi: 10.3389/fnagi.2022.995425 (PMC9581239; doi:10.3389/fnagi.2022.995425)
Supplement: Supplementary file 1 [file Data_Sheet_1.docx]

**Supplementary Tables and Supplementary Figure Captions**

| Table S1 |  |  |  |  | |
| --- | --- | --- | --- | --- | --- |
|  |  |  | **Sequences** |  | |
| Parameters | **T1** | **Gradient-Echo BOLD**  **Sequence** | **Gradient-Echo BOLD Fieldmap** | **Spin-Echo**  **Multi-Shell Diffusion** | **Reverse-Phase Encoding Sequence** |
| Field of View | 256 x 256 x 176  mm^3^ | 192 x 192 x120  mm^3^ | 192 x 192 x120  mm^3^ | 232 x 232 x 162  mm^3^ | 232 x 232 x162  mm^3^ |
| Voxel Size | 1 mm^3^ | 3 mm^3^ | 3 mm^3^ | 2 mm^3^ | 2 mm^3^ |
| Repetition Time | 2530 ms | 2500 ms | 450 ms | 3400 ms | 3400 ms |
| Echo Time(s) | 1.69, 3.55, 5.41, and 7.27 ms | 30 ms | 5.19 and 7.65 ms | 71 ms | 71 ms |
| Flip Angle | 7 degrees | 90 degrees | 60 degrees | 90, then 180 degrees | 90, then 180 degrees |
| Acquisition Time | 5.88 minutes | 4.12 minutes | 1.23 minutes | 7.45 minutes | 0.47 minutes |
| Acceleration Type/Factor | GRAPPA, Factor 2 | Off | Off | Multislice Acceleration, Factor 3 | Multislice Acceleration, Factor 3 |
| Phase Partial Fourier | Off | Off | Off | 6/8 | 6/8 |
| b-Values | N/A | N/A | N/A | 0, 500, 1000, 2000 s/mm^2^ | 0 and 2000 s/mm^2^ |

**Table S1.** Summary of MRI Sequences. Columns correspond to the sequences employed, while rows correspond to sequence parameters. BOLD = Blood-Oxygen Level-Dependent, GRAPPA = Generalized Autocalibrating Partial Parallel Acquisition, mm = millimeters, ms = milliseconds.

| Table S2 |  | |  |  | Age |  |  |
| --- | --- | --- | --- | --- | --- | --- | --- |
|  | **Top 2%** | |  | **Top 5%** |  | **Top 10%** |  |
| Diffusion Metric | | Standardized Beta | *p*-value | Standardized Beta | *p*-value | Standardized Beta | *p*-value |
| Model 1 |  | |  |  |  |  |  |
| FA | -0.414 | | < 0.001* | -0.421 | < 0.001* | -0.400 | < 0.001* |
| Model 2 |  | |  |  |  |  |  |
| FW | 0.062 | | 0.604 | 0.051 | 0.678 | 0.065 | 0.605 |
| FWE-FA | -0.404 | | 0.001* | -0.421 | 0.001* | -0.393 | 0.002* |
| Model 3 |  | |  |  |  |  |  |
| ICVF | -0.369 | | < 0.001* | -0.385 | < 0.001* | -0.400 | < 0.001* |
| ODI | 0.238 | | 0.015* | 0.249 | 0.010* | 0.224 | 0.021* |
| CSF Fraction | 0.122 | | 0.197 | 0.135 | 0.152 | 0.133 | 0.160 |

**Table S2.** Summary of Linear Regression Models with Diffusion Metrics Predicting Age using Differently Thresholded Working Memory Network White Matter Masks. The results are essentially the same regardless of the threshold implemented. Sex was used as a covariate in all models. **p* < 0.05

| Table S3 |  | |  |  | D-Prime |  |  |
| --- | --- | --- | --- | --- | --- | --- | --- |
|  | **Top 2%** | |  | **Top 5%** |  | **Top 10%** |  |
| Diffusion Metric | | Standardized Beta | *p*-value | Standardized Beta | *p*-value | Standardized Beta | *p*-value |
| Model 1 |  | |  |  |  |  |  |
| FA | 0.189 | | 0.082 | 0.165 | 0.128 | 0.142 | 0.185 |
| Model 2 |  | |  |  |  |  |  |
| FW | 0.059 | | 0.633 | 0.048 | 0.710 | 0.059 | 0.651 |
| FWE-FA | 0.285 | | 0.033* | 0.253 | 0.066 | 0.241 | 0.078 |
| Model 3 |  | |  |  |  |  |  |
| ICVF | 0.249 | | 0.020* | 0.241 | 0.027* | 0.228 | 0.037* |
| ODI | -0.099 | | 0.343 | -0.054 | 0.603 | -0.030 | 0.775 |
| CSF Fraction | 0.051 | | 0.609 | 0.051 | 0.616 | 0.057 | 0.575 |

**Table S3.** Summary of Linear Regression Models with Diffusion Metrics Predicting Working Memory Performance using Differently Thresholded Working Memory Network White Matter Masks. The results are similar regardless of the threshold implemented. Age and sex were used as covariates in all models. **p* < 0.05

| Table S4 |  | Age |  |
| --- | --- | --- | --- |
| Diffusion Metric | Standardized Beta | t-value | *p*-value |
| Model 1 |  |  |  |
| FA | -0.425 | -4.518 | < 0.001* |
| Model 2 |  |  |  |
| FWE-FA | -0.452 | -4.862 | < 0.001* |
| Model 3 |  |  |  |
| FW | 0.328 | 3.370 | 0.001* |
| Model 4 |  |  |  |
| ICVF | -0.360 | -3.760 | < 0.001* |
| Model 5 |  |  |  |
| ODI | 0.191 | 1.865 | 0.065 |
| Model 6 |  |  |  |
| CSF Fraction | 0.166 | 1.631 | 0.106 |

**Table S4.** Summary of Linear Regression Models with Diffusion Metrics Extracted from the WMN-WM Mask Predicting Age Using A 6 Model Approach. The results are very similar as the main findings, except that FW was strongly associated with age when FWE-FA was not included in the same model. **p* < 0.05

| Table S5 |  | D-Prime |  |
| --- | --- | --- | --- |
| Diffusion Metric | Standardized Beta | t-value | *p*-value |
| Model 1 |  |  |  |
| FA | 0.185 | 1.724 | 0.088 |
| Model 2 |  |  |  |
| FWE-FA | 0.241 | 2.229 | 0.028* |
| Model 3 |  |  |  |
| FW | -0.099 | -0.955 | 0.342 |
| Model 4 |  |  |  |
| ICVF | 0.226 | 2.206 | 0.030* |
| Model 5 |  |  |  |
| ODI | -0.036 | -0.350 | 0.727 |
| Model 6 |  |  |  |
| CSF Fraction | 0.035 | 0.346 | 0.730 |

**Table S5.** Summary of Linear Regression Models with Diffusion Metrics Extracted from the WMN-WM Mask Predicting Working Memory Performance Using A 6 Model Approach. The results are extremely similar as the main findings. **p* < 0.05

**Supplementary Figure Captions**

**Figure S1.** A 3D display of the Working Memory Network White Matter Mask Calculated using Probabilistic Tractography. The figure displays a 3-dimensional rendering of the calculated group-level WMN-WM tracts using probabilistic tractography (red; *section 2.7.1*). Peak regions of activation during the working memory tasks [(2-back/2 + 1-back/2) > Compare] were used as seed regions (Yellow anterior regions; Blue, posterior regions) to create this WMN-WM mask.

**Figure S2.** Differently Thresholded Group Working Memory Network White Matter Masks Calculated using Probabilistic Tractography. The figure displays differently thresholded group-level-calculated white matter tracts overlaid onto a 1mm MNI152 template. The thresholds are displayed such that the most lenient threshold is placed first (top 10% of all voxels, yellow) and subsequent more stringent thresholds are placed on top of each other (top 5%, green; top 3%, red, threshold used in study; top 2%, blue).

**Figure S3.** Q-Q Plots of Standardized Error Residuals. The figure displays the Q-Q plots of the standardized error residuals from each of the linear regression models predicting age (left column) and D-prime (right column). Error residuals in all linear regression models follow a normal distribution.
