# Supplementary figures and images for: Multi-compartment diffusion magnetic resonance imaging models link tract-related characteristics with working memory performance in healthy older adults
Source: Front Aging Neurosci. 2022 Oct 5;14:995425. doi: 10.3389/fnagi.2022.995425 (PMC9581239; doi:10.3389/fnagi.2022.995425)

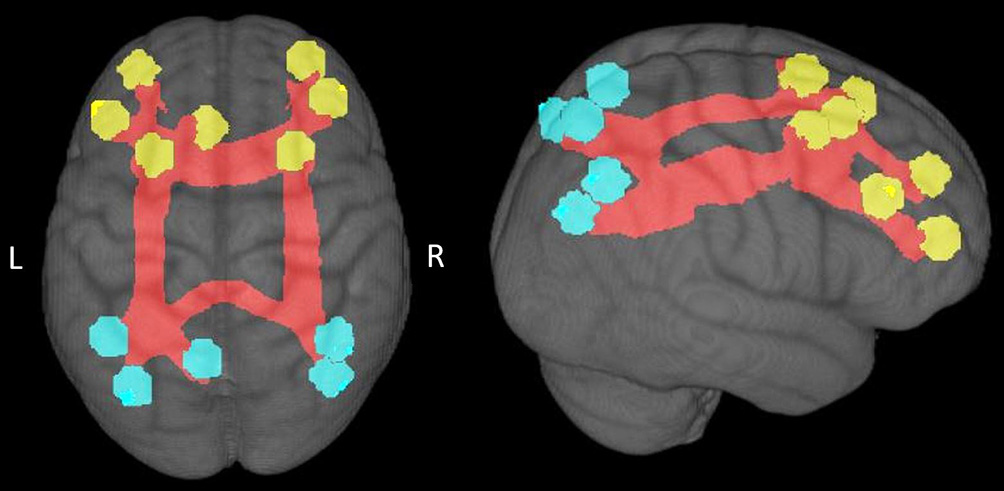

Supplement: Supplementary file 2 [file Image_1.jpg]

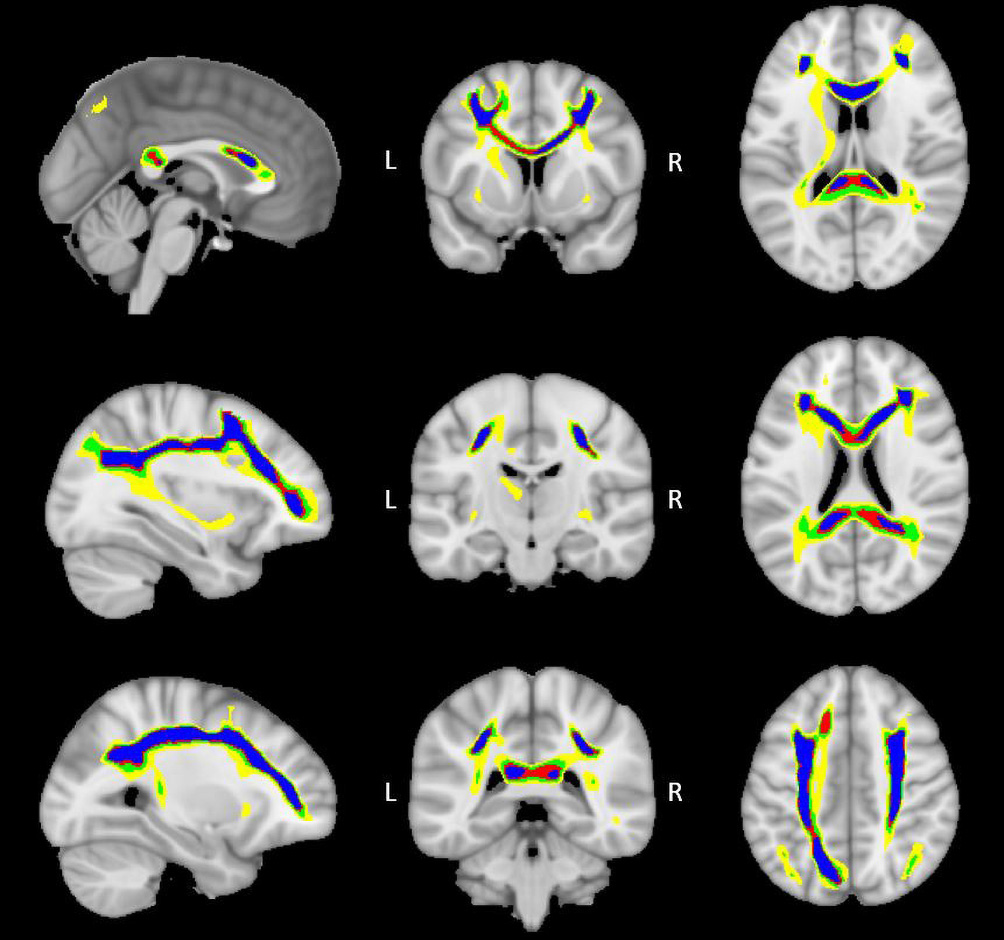

Supplement: Supplementary file 3 [file Image_2.jpg]

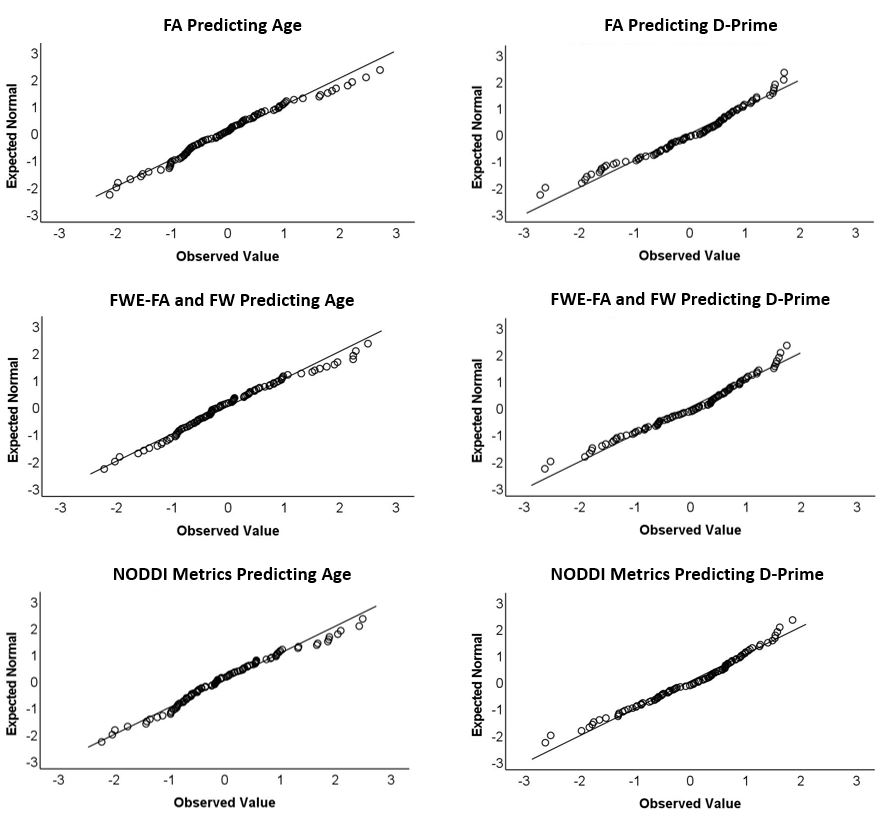

Supplement: Supplementary file 4 [file Image_3.jpg]
